# A Lightweight Double Compression Detector for HEIF Images Based on Encoding Information

**DOI:** 10.3390/s24165103

**Published:** 2024-08-06

**Authors:** Yoshihisa Furushita, Marco Fontani, Stefano Bianchi, Alessandro Piva, Giovanni Ramponi

**Affiliations:** 1 Department of Information Engineering, University of Florence, 50139 Florence, Italy; yoshihisa.furushita@unifi.it; 2 Amped Software, 34149 Trieste, Italy; marco.fontani@ampedsoftware.com (M.F.);; 3 Dipartimento di Ingegneria e Architettura (DIA), Università Degli Studi di Trieste, 34127 Trieste, Italy

**Keywords:** image forensics, double compression, HEIF, coding ghosts

## Abstract

Extensive research has been conducted in image forensics on the analysis of double-compressed images, particularly in the widely adopted JPEG format. However, there is a lack of methods to detect double compression in the HEIF format, which has recently gained popularity since it allows for reduced file size while maintaining image quality. Traditional JPEG-based techniques do not apply to HEIF due to its distinct encoding algorithms. We previously proposed a method to detect double compression in HEIF images based on Farid’s work on coding ghosts in JPEG images. However, this method was limited to scenarios where the quality parameter used for the first encoding was larger than for the second encoding. In this study, we propose a lightweight image classifier to extend the existing model, enabling the identification of double-compressed images without heavily depending on the input image’s quantization history. This extended model outperforms the previous approach and, despite its lightness, demonstrates excellent detection accuracy.

## 1. Introduction

Today’s widespread availability of mobile devices for capturing visual data means that almost everyone can easily record, store, and share vast quantities of digital images. Simultaneously, the abundance of image editing tools makes modifying or creating images incredibly simple, making the manipulation and falsification of visual content no longer limited to experts. Consequently, manipulated images are becoming more prevalent across various fields, eroding the trustworthiness of visual content. To address this issue, the research community has developed the image forensics discipline. This field relies on the idea that every stage of the image lifecycle, such as acquisition, compression, and editing, leaves traces in the image data [1]. By detecting these traces, it becomes possible to trace the origin of an image and verify its integrity.

As the JPEG format [2] has been widely used in most digital cameras and image processing tools for decades, much of the image forensics research has addressed this class of images. In particular, starting from the hypothesis that manipulation is obtained by reading a JPEG image, editing it, and saving it again in JPEG format, one of the most used solutions to detect tampering is examining the artifacts left during JPEG recompression. These artifacts can be classified into two categories: aligned double JPEG (A-DJPG) compression, in which the discrete cosine transform (DCT) grid of the first and second JPEG compressions align, and non-aligned double JPEG (NA-DJPG) compression, in which they do not align. Research in A-DJPG compression has explored the double quantization (DQ) effect that alters the histogram of DCT coefficients [3,4], or Benford’s law [5,6,7], and leveraged the idempotence of quantization [8]. On the other hand, studies on NA-DJPG compression observed changes in the regularity of block artifacts [9,10,11,12] and clustering patterns of DCT coefficients [13].

JPEG was the de facto standard for digital images, but its limitations became apparent as video and display technology advanced. This led to a growing demand for a compression method that delivers smaller file sizes without compromising image quality. In 2017, Apple introduced the HEIF format, which offers twice the compression efficiency of JPEG while preserving image quality [14]. The HEIF standard allows for multiple data compression methods, the most popular of which is H.265/HEVC, originally designed for video encoding and used in HEIF to compress individual images. As of July 2024, Apple’s iOS is one of the major users of HEVC, with an approximately 30% share of the global mobile OS market (https://gs.statcounter.com/os-market-share/mobile/worldwide, accessed on 1 August 2024). Android has the largest market share and has supported HEIF since version 10. Adobe Photoshop also offers HEIF editing capabilities.

Although HEIF is considered a potential successor to JPEG, research on HEIF images in the context of digital forensics is limited [15,16], and even more restricted on HEIF double compression detection [17]. This is probably because JPEG and HEIF standards use different encoding techniques, compression algorithms, and formats; therefore, directly applying JPEG double compression detection methods to HEIF images may not yield effective results.

On the other hand, research on double compression detection in HEVC videos, which use the same encoding technology as HEIF images, has attracted attention. A video consists of several frames, including P-frames predicted from past frames, B-frames predicted from both past and future frames, and I-frames that use spatial correlation to predict neighboring pixels and are coded independently of neighboring frames. A continuous group of frames starting with an I-frame and consisting of P- or B-frames is called a Group of Pictures (GOP).

In practical scenarios, an I-frame may be re-encoded using a GOP of a different length than the original and encoded as a P-frame. This frame, known as a relocated I-frame, breaks the temporal and spatial consistency in the GOP and serves as a clue for detecting double compression [18,19,20]. Other studies focus on changes in quantized DCT coefficients [21] and differences in encoded elements within P-frames [22] between single-compressed and double-compressed videos.

When a video is re-encoded using the same quality parameter, the encoding history is overwritten, making double compression detection difficult. Jiang et al. found that changes in encoded elements within I-frames are most significant between single-compressed and double-compressed videos and tend to remain stable with additional compressions, aiding the detection of double compression [23].

These previous works on double compression detection in HEVC videos can provide useful insights for research on double compression detection in HEIF images. However, still images contain only a single frame and cannot utilize temporal correlation like videos. Therefore, detecting double compression in HEIF images must rely on spatial correlation only. Additionally, there are limitations on the feature vectors that can be extracted from the dataset, resulting in fewer materials available for input into the double compression classifier.

This work extends our previous research [17], which took inspiration from Farid’s work [8] to detect double compression in HEIF images. Ref. [17] was limited to cases where the quantization parameter (QP) used in the first encoding was larger than in the second, and performance degraded when the difference between the first and second QP was smaller than 5. In this study, we focus on the fact that the change in the encoding factors between the input image and its recompressed image depends on the compression history of the input image. By incorporating these statistical features as a new feature vector fed to a support vector machine, we aim to detect double compression without being excessively hampered by the encoding history of the input image.

This work contributes to image forensics, particularly in the context of double compression detection in HEIF images. The key contributions of this paper are as follows:

- Our work is the first study to address double compression in HEIF images and extends the findings presented in [17]. It effectively addresses the weaknesses identified in the initial work and provides a more robust and comprehensive analysis.
- We have developed a robust method for detecting double compression, even when images are encoded with various combinations of parameters.

This paper is structured as follows: Section 2 provides an overview of the H.265/HEVC architecture. Section 3 outlines Farid’s method for identifying double compression in JPEG images. Section 4 describes our proposed method, and Section 5 presents the experiments and results.

## 2. Overview of H.265/HEVC

This section explains the basic technology of the H.265/HEVC encoding standard [24,25]. However, since this study focuses on still images, we omit the explanation of the techniques used only for video data.

### 2.1. Characteristics of Encoding Units

**Figure 1:**
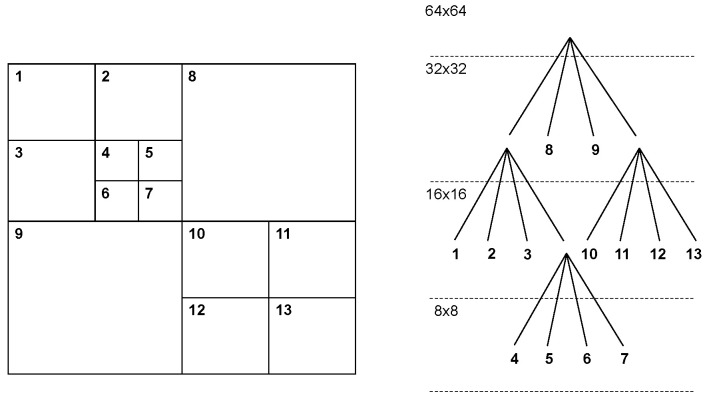
Example of the partitioning of 64 × 64 CTU into various size of CU.

In H.265/HEVC, an image is divided into blocks for efficient encoding processing using four processing units as follows: Coding Tree Unit (CTU), Coding Unit (CU), Prediction Unit (PU), and Transform Unit (TU). The smallest image partitioning and basic encoding units are the CTU and CU, respectively. Each CTU consists of Coding Tree Blocks (CTB) for luminance and chrominance components and is further divided into variable-size CUs based on recursive quad-tree partitioning, as described in Figure 1. Each CU consists of Coding Blocks (CB) for luminance and chrominance components. Furthermore, each CU is divided into variable-size PUs and TUs based on recursive quad-tree partitioning, each responsible for prediction and transformation processing. Table 1 shows the maximum and minimum sizes of the four processing blocks. The introduction of CTU, CU, PU, and TU permits the encoder’s tailoring to the image’s characteristics and minimizes prediction parameters, thus reducing encoding costs. For example, in regions of the image with complex changes, many small CUs allocate more prediction parameters, such as motion vectors, improving prediction performance. Conversely, large CUs are used for encoding in regions with few changes.

### 2.2. Intra-Prediction

**Figure 2:**
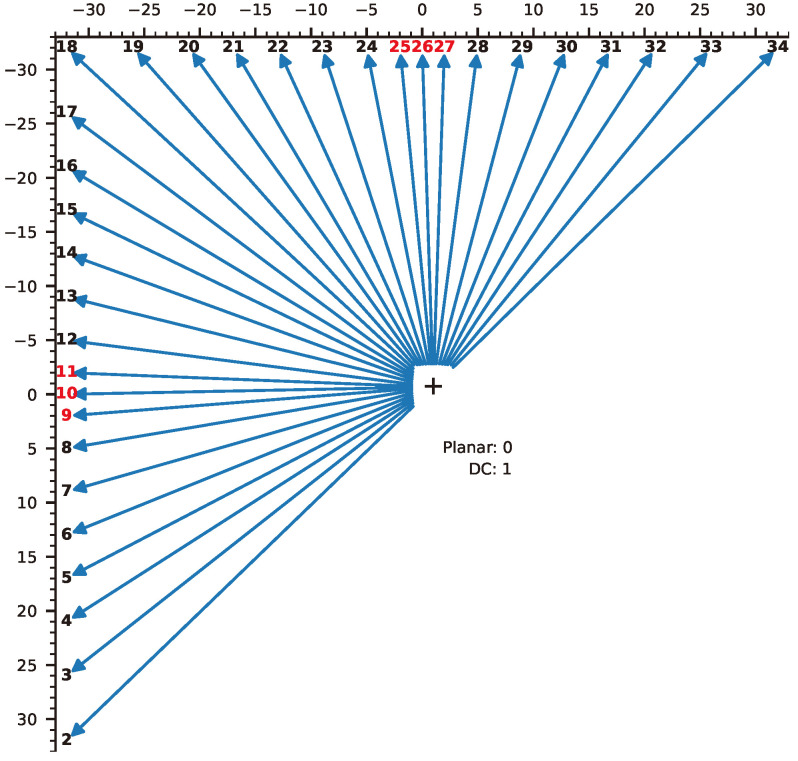
Intra-prediction mode for luminance components.

In H.265/HEVC, intra-prediction is performed on luminance and color-difference signals to reduce redundancy and increase compression efficiency by taking advantage of the high correlation between adjacent pixels in an image. The prediction mode is signaled at the PU level, while the encoding, decoding, and prediction processes are performed at the TU level. As described in Figure 2, intra-prediction for the luminance component uses two standard prediction operators (DC and Planar) and 33 oriented (angular) operators. Angular operators (2–34) predict a target pixel referring to an encoded pixel in the specified angles. Planar prediction (0) uses interpolated values from four adjacent pixels, while DC prediction (1) uses the average value of surrounding pixels. Each PU is assigned an intra-prediction mode, and processing is performed at the TU level. The angular displacements of the prediction modes closer to the horizontal and vertical directions are set smaller than in other directions because natural images contain more almost horizontal and almost vertical patterns than in other directions, as shown in red in the figure. Intra-prediction for chrominance components employs planar (0), DC (1), horizontal (10), vertical (26), and intra-derived modes (36). The planar, DC, horizontal, and vertical prediction modes are explicitly signaled, but if they match the luminance intra-prediction mode, the angular prediction mode (34) is applied instead. In the intra-derived mode (36), chrominance intra-prediction uses the corresponding luminance intra-prediction mode to reduce signal overhead for encoding.

## 3. Coding Ghosts in JPEG Images

Our research is inspired by previous studies on JPEG compression idempotency, which Farid proposed [8]. Compression idempotency means that when an original image is compressed repeatedly with the same encoding parameters, the resulting compressed image remains close to the original in terms of visual quality and characteristics.

Idempotency can be expressed as follows [26]: In JPEG compression, a color image transforms luminance (Y) and chrominance (Cb and Cr) channels, partitioning into 8 × 8 pixel blocks. These blocks are then subjected to Discrete Cosine Transform (DCT), converting the image data from spatial to frequency domains. The frequency domain components (i.e., the DCT coefficients) of the input image $\left(I_{fd}\right)$ are quantized using a quantizer *Q* with step size $\Delta_{1}$ ($Q_{\Delta_{1}}$). This process involves dividing each frequency component by $\Delta_{1}$ and rounding ($\left\lfloor .\right\rfloor$) the result to derive $I_{fd}^{\prime }$.

(1) $$I_{fd}^{\prime }=Q_{\Delta_{1}}\left(I_{fd}\right)=\Delta_{1}\left\lfloor \frac{I_{fd}}{\Delta_{1}}\right\rfloor \;,$$

Let us note that the quantization step size ($\Delta_{1}$) is determined by a quality factor (QF). Higher QF values yield smaller $\Delta_{1}$, preserving image quality, while lower QF values lead to larger $\Delta_{1}$, facilitating image compression at the expense of quality. Suppose now that the compressed image is re-compressed, using the quantization parameter $Q_{\Delta_{2}}$. The frequency domain components $I_{fd}^{\prime \prime }$ are obtained. Idempotency consists in that if the same quantization parameter is used in the two compression processes (i.e., $\Delta_{2}=\Delta_{1}$), $I_{fd}^{\prime \prime }$ will be equal to $I_{fd}^{\prime }$.

(2) $$I_{fd}^{\prime \prime }=Q_{\Delta_{2}}\left(I_{fd}^{\prime }\right)=\Delta_{2}\left\lfloor \frac{\Delta_{1}\left\lfloor \frac{I_{fd}^{\prime }}{\Delta_{1}}\right\rfloor }{\Delta_{2}}\right\rfloor \;=\Delta_{1}\left\lfloor \frac{\Delta_{1}\left\lfloor \frac{I_{fd}^{\prime }}{\Delta_{1}}\right\rfloor }{\Delta_{1}}\right\rfloor \;=I_{fd}^{\prime }.$$

We applied this idea to HEIF images since the compression processes in JPEG and HEIF are based on similar concepts.

Consider $D_{0}$ as the collection of DCT coefficients from an image $I_{0}$, quantized with $Q_{0}$ as a QF. Let us assume that an uncompressed image, denoted by *I*, undergoes subsequent compression with a different QF, $Q_{1}$, resulting in the coefficients set $D_{1}$. Farid’s research [8] demonstrated that the disparity between $D_{0}$ and $D_{1}$ is minimized when $Q_{0}=Q_{1}$, owing to the idempotent nature of JPEG compression.

This idempotency forms the basis for verifying double compression. Starting with an uncompressed image (*I*) compressed initially at $Q_{0}$, followed by a second compression at $Q_{1}$ (assuming $Q_{0}<Q_{1}$) to obtain image $I_{1}$, the resulting image data include DCT coefficients quantized with both $Q_{1}$ and $Q_{0}$. For experimental verification, the image $I_{1}$ undergoes recompression using a quantization value $Q_{2}$, yielding the image $I_{2}$ with a corresponding set of DCT coefficients $D_{2}$. As previously discussed, the discrepancy between $D_{1}$ and $D_{2}$ is minimized when $Q_{2}$ is the same as $Q_{1}$. However, considering that $D_{2}$ encompasses data initially quantized with $q_{0}$, an additional minimum occurs when $Q_{2}$ is the same as $Q_{0}$. This additional minimum is commonly termed the “JPEG ghost”.

**Figure 3:**
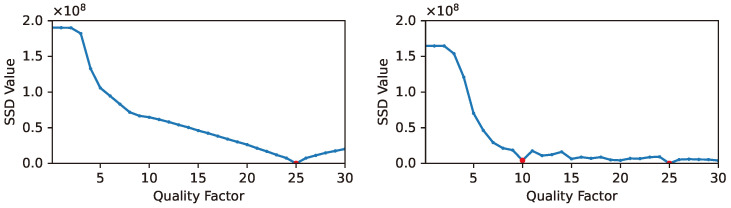
Sum of squared differences (SSD) vs. quality factor. (**Left**: Single-compressed image; **Right**: Double-compressed image).

The behavior of the JPEG ghost with varying quantization step sizes is illustrated in Figure 3. In the left figure, we observe the sum of squared differences (SSD) among coefficients quantized using a step size of $Q_{1}=25$, followed by a subsequent quantization within the range $Q_{2}\in \left[1,30\right]$. The minimal difference occurs precisely when $Q_{2}=Q_{1}=25$. In the right figure, we examine the SSD among coefficients initially quantized at $Q_{0}=10$, then $Q_{1}=25$, and further quantized within the same range $Q_{2}\in \left[1,30\right]$. Here, the minimum discrepancy arises at $Q_{2}=Q_{1}=25$, accompanied by a local minimum at $Q_{2}=Q_{0}=10$. In Farid’s analysis, this comparison can also be conducted in the spatial domain using RGB pixel values. By identifying re-compressed versions with the smallest variances, we can detect double compression by observing the presence of the JPEG ghost.

The attack described in Farid’s research involves an attacker copying tampered regions from another JPEG image and pasting them into a target image. Our study generalizes this approach by using double-compressed images rather than tampered ones. Since tampering implies that the image has been decoded and re-encoded, we focus on detecting entire images affected by double compression rather than specific regions copied from other JPEG images.

## 4. Proposed Method

Ref. [17] detected double compression in HEIF images by computing two mean absolute error (MAE) differences. One compared the input image with its recompressed version at varying QP, while the other compared the calibrated input image (an image that has been adjusted to minimize the influence of its visual content) with its recompressed version at the same QP. We successfully identified double compression by finding the difference between these vectors and applying a detection rule.

Calibration was employed to ensure that the shape of the different plots was not influenced by the visual content of the input image. For instance, the sky in an image is generally uniform, with little variation between pixels, leading to smaller pixel differences and less noticeable local variations. To address this, we converted the image (1200 × 800) to a NumPy array of RGB channels and shifted each row of RGB pixels by 15 pixels to the right. This adjustment allowed us to generate two MAE curves for the input image, accurately capturing local MAE variations. This technique has been proven effective in prior studies [27,28].

However, the method of [17] only works when the QP used for the first encoding ($QP_{1}$) is larger than the QP used for the second encoding ($QP_{2}$). Theoretically, if $QP_{1}$ is the same as $QP_{2}$, no coding ghosts occur. Also, the experimental validation shows that for the case $QP_{1}$ is smaller than $QP_{2}$, HEIF ghosts do not emerge.

**Figure 4:**
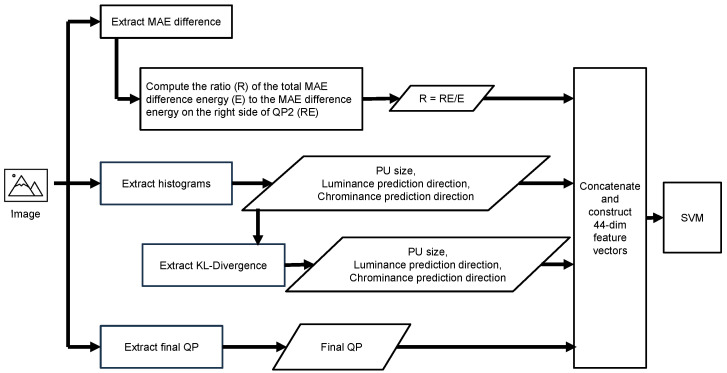
Flowchart of the proposed method.

To ensure that double-compressed images can be detected without depending heavily on the compression history of the input image, we have added to the feature vector the change in encoding elements between the image and its recompressed version. The flowchart of the proposed method is shown in Figure 4. This flowchart visually represents the process of feature vector extraction involved in detecting double compression of HEIF images.

In the subsequent sections, after outlining our existing approach for detecting double compression in HEIF images, we discuss the changes occurring in encoding elements between the input image and its recompressed version. We also detail the new feature vector and the SVM classifier used in our research. The proposed method assumes that the QP used in the last encoding of the input image is known. This assumption is easily fulfilled since the QP value used in the last encoding can be identified by just examining the file header.

### 4.1. MAE Difference Extraction and the Analysis

Let *I* denote a single- or double-compressed input image. Our method starts by recompressing *I* with varying QP values from 1 to 51, resulting in 51 recompressed images labeled as ${\hat{I}}_{1},{\hat{I}}_{2},...,{\hat{I}}_{51}$. For each recompressed image, we compute its mean absolute error (MAE) against the original input image using the following equation, where (x,y) represents pixel coordinates within images of dimensions *W* (width) and *H* (height):

(3) $$MAE\left(I,{\hat{I}}_{QP}\right)=\frac{1}{WH}\sum_{x=0}^{W-1}\sum_{y=0}^{H-1}\left|I\left(x,y\right)-{\hat{I}}_{QP}\left(x,y\right)\right|.$$

**Figure 5:**
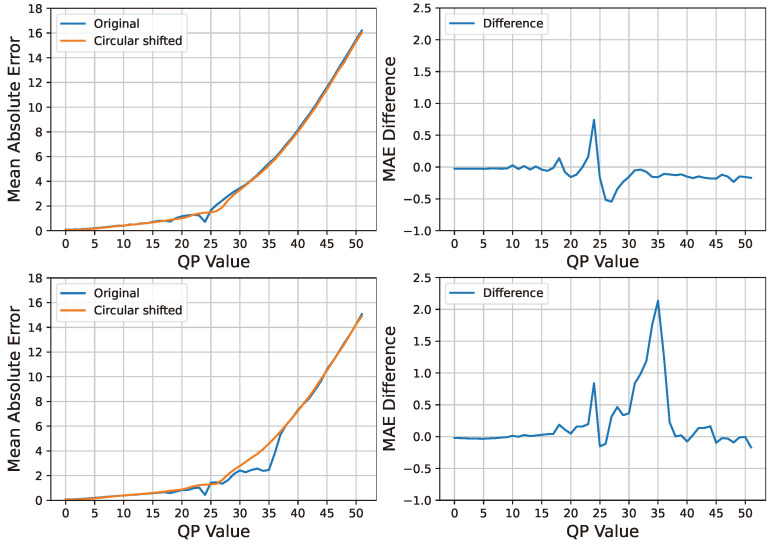
MAE curves and their MAE differences at each QP. (**Top**: QP = 24; **Bottom**: $QP_{1}$ = 35, $QP_{2}$ = 24).

Next, let CI represent the input image after circular shifting. We perform recompression on CI using QP values ranging from 1 to 51, resulting in 51 recompressed images labeled as ${\hat{CI}}_{1},{\hat{CI}}_{2},...,{\hat{CI}}_{51}$. Similarly, we compute the MAE between each recompressed circular shifted image and the original input image for each QP value. Figure 5 illustrates an example of the MAE curves and their MAE differences for a single- and double-compressed image. The upper figures show an image encoded with QP=24, while the lower figures show that it is first encoded with $QP_{1}=35$ and then recompressed with $QP_{2}=24$. The left side of the figure displays the original MAE curve and the circular shifted MAE curve for the input image, while the right side presents the MAE differences at each QP value. The upper image shows a maximum MAE difference of 24, indicating a single compressed image. On the other hand, in the lower image, the MAE difference is observed to be larger at 24 and 35 than the other QP values on the *x*-axis, suggesting that the input image is double-compressed.

(4) $$MAE\left(CI,{\hat{CI}}_{QP}\right)=\frac{1}{WH}\sum_{x=0}^{W-1}\sum_{y=0}^{H-1}\left|CI\left(x,y\right)-{\hat{CI}}_{QP}\left(x,y\right)\right|.$$

According to these results, we detected double compression in HEIF images by analyzing the MAE difference plot. As explained earlier, when an image undergoes double compression, an additional peak should appear to the right of $QP_{2}$, as illustrated in Figure 5. In contrast, no additional peak appears to the right of the last QP value for single compression. We represent the MAE differences for each QP value as an array, denoted by $M=[M_{1},M_{2},M_{3},\ldots ,M_{51}]$. Detecting double compression involves comparing the ratio of the total MAE difference energy (denoted as $E=\sum_{i=1}^{51}{\left(M_{i}\right)}^{2}$) to the MAE difference energy in the right-hand portion of $QP_{2}$ (denoted as $RE=\sum_{i=QP_{2}+1}^{51}{\left(M_{i}\right)}^{2}$), that is for all QP values higher than $QP_{2}$. In our new approach, the ratio ($R=\frac{RE}{E}$) serves as one of the image’s feature vectors without setting a specific threshold.

### 4.2. Statistical Analysis for Changes of Encoding Elements between Images

HEVC encodes images on a block-by-block basis, and the selection of the PU size and prediction mode can vary between compression cycles due to quantization errors and rate distortion (RD) cost optimization. Previous research on HEVC video suggests that the change in PU size between I-frames of single-compressed and double-compressed images is larger than that between double-compressed and triple-compressed images at the same QP [23].

We utilized the Kullback–Leibler Divergence (KLD) to compare the changes in encoding elements between input images and their recompressed counterparts. KLD is particularly useful in this context as it measures the distance between two probability distributions, allowing us to quantify the difference in encoding element distributions caused by different compression cycles. This helps identify the compression artifacts and discrepancies that are more pronounced in double compression than in single compression.

To obtain the KLD values, we used the open-source bitstream converter heic2hevc [29] to convert the input and recompressed images into HEVC bitstream. The bitstream was then decoded using the HM 16.25 [30] to extract information on PU sizes (64 × 64, 32 × 32, 16 × 16, 8 × 8, 4 × 4), luminance prediction directions (0, 1, 9, 10, 11, 25, 26, 27), and chrominance prediction directions (0, 1, 10, 26, 34, 36) for each 4 × 4 block. We computed histograms for each encoding element based on the information extracted for each 4 × 4 block. Laplace smoothing with a parameter α=1 was applied to avoid zero probabilities in the probability distribution. The smoothed probabilities $\hat{p}$ for an input image and $\hat{q}$ for its recompressed image are calculated as follows:

(5) $$\hat{p_{i}}=\frac{C_{p,i}+\alpha }{\sum_{j=1}^{N}C_{p,j}+\alpha N},\;\hat{q_{i}}=\frac{C_{q,i}+\alpha }{\sum_{j=1}^{N}C_{q,j}+\alpha N}\;\left(i=1,2,3,\ldots ,N\right).$$

where $C_{p,i}$ and $C_{q,i}$ are the aggregate number of coding elements in each category, $\sum_{j=1}^{N}C_{p,j}$ and $\sum_{j=1}^{N}C_{q,j}$ are the corresponding total aggregate number of coding elements, and *N* is the number of categories.

The KL divergence between probability distributions *P* and *Q* is given by:

(6) $$KL\left(P\|Q\right)=\sum_{i=1}^{N}\hat{p_{i}}\log\left(\frac{\hat{p_{i}}}{\hat{q_{i}}}\right)\;\left(i=1,2,3,\ldots ,N\right).$$

Thus, for PU sizes (64 × 64, 32 × 32, 16 × 16, 8 × 8, 4 × 4), luminance prediction directions (0, 1, 9, 10, 11, 25, 26, 27), and chrominance prediction directions (0, 1, 10, 26, 34, 36), the KL divergences are computed as follows:

1. PU sizes (five categories):
  (7) $${\hat{p}}_{PU_{i}}=\frac{C_{p_{PU,i}}+1}{\sum_{j=1}^{5}C_{p_{PU,j}}+5},\;{\hat{q}}_{PU_{i}}=\frac{C_{q_{PU,i}}+1}{\sum_{j=1}^{5}C_{q_{PU,j}}+5}\;\left(i=1,2,3,4,5\right),$$
  (8) $$KL\left(P_{PU}\|Q_{PU}\right)=\sum_{i=1}^{5}{\hat{p}}_{PU_{i}}\log\left(\frac{{\hat{p}}_{PU_{i}}}{{\hat{q}}_{PU_{i}}}\right).$$
2. Luminance prediction direction (eight categories):
  (9) $${\hat{p}}_{LUMA_{i}}=\frac{C_{p_{LUMA,i}}+1}{\sum_{j=1}^{8}C_{p_{LUMA,j}}+8},\;{\hat{q}}_{LUMA_{i}}=\frac{C_{q_{LUMA,i}}+1}{\sum_{j=1}^{8}C_{q_{LUMA,j}}+8}\;\left(i=1,2,3,4,5,6,7,8\right),$$
  (10) $$KL\left(P_{LUMA}\|Q_{LUMA}\right)=\sum_{i=1}^{8}{\hat{p}}_{LUMA_{i}}\log\left(\frac{{\hat{p}}_{LUMA_{i}}}{{\hat{q}}_{LUMA_{i}}}\right).$$
3. Chrominance prediction direction (six categories):
  (11) $${\hat{p}}_{CHROMA_{i}}=\frac{C_{p_{CHROMA,i}}+1}{\sum_{j=1}^{6}C_{p_{CHROMA,j}}+6},\;{\hat{q}}_{CHROMA_{i}}=\frac{C_{q_{CHROMA,i}}+1}{\sum_{j=1}^{6}C_{q_{CHROMA,j}}+6}\;\left(i=1,2,3,4,5,6\right),$$
  (12) $$KL\left(P_{CHROMA}\|Q_{CHROMA}\right)=\sum_{i=1}^{6}{\hat{p}}_{CHROMA_{i}}\log\left(\frac{{\hat{p}}_{CHROMA_{i}}}{{\hat{q}}_{CHROMA_{i}}}\right).$$

**Figure 6:**
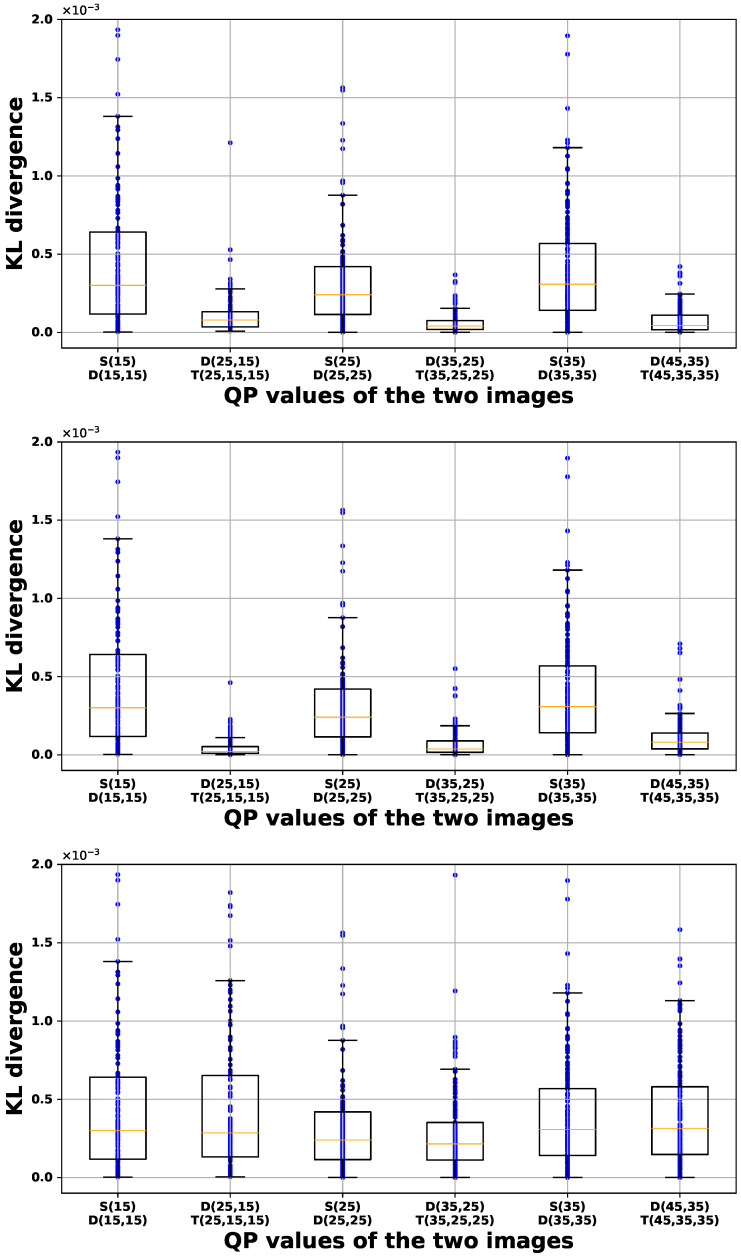
Box-and-whisker diagram of KL divergence for PU size. (**Top**: $QP_{1}>QP_{2}$; **Middle**: $QP_{1}=QP_{2}$; **Bottom**: $QP_{1}<QP_{2}$).

**Figure 7:**
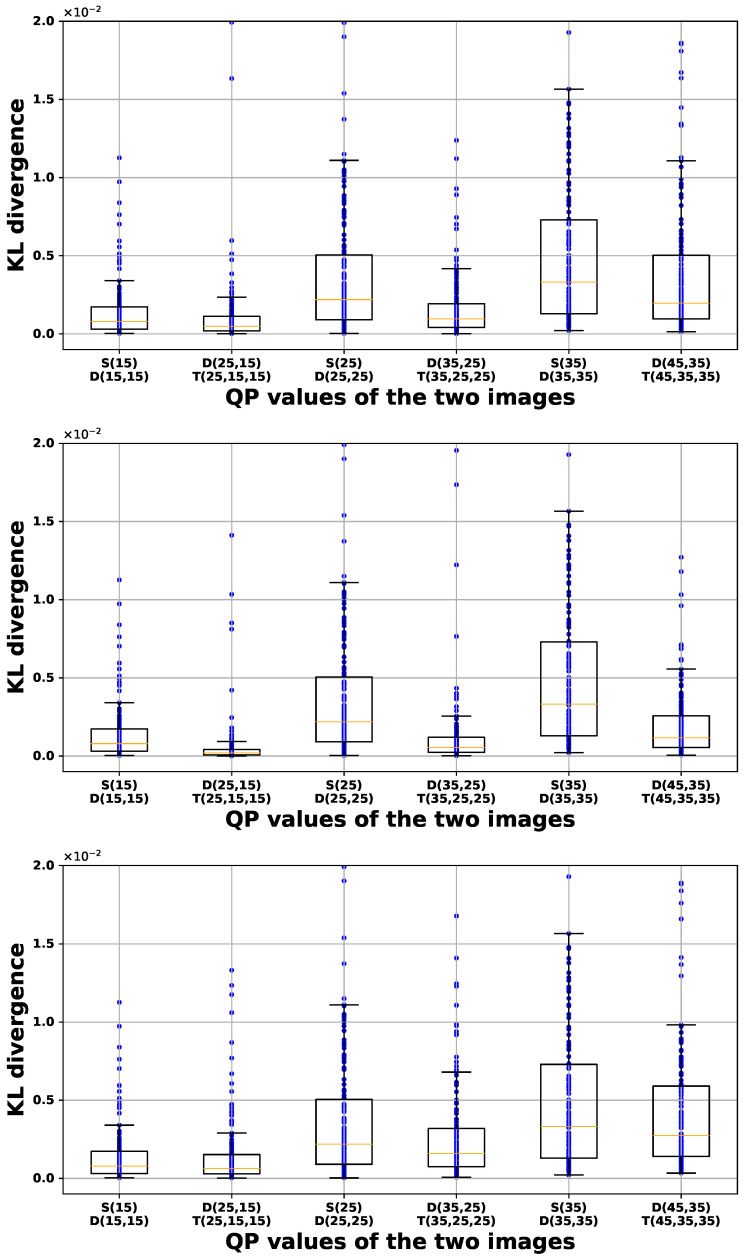
Box-and-whisker diagram of KL divergence for luminance intra-prediction direction. (**Top**: $QP_{1}>QP_{2}$; **Middle**: $QP_{1}=QP_{2}$; **Bottom**: $QP_{1}<QP_{2}$).

**Figure 8:**
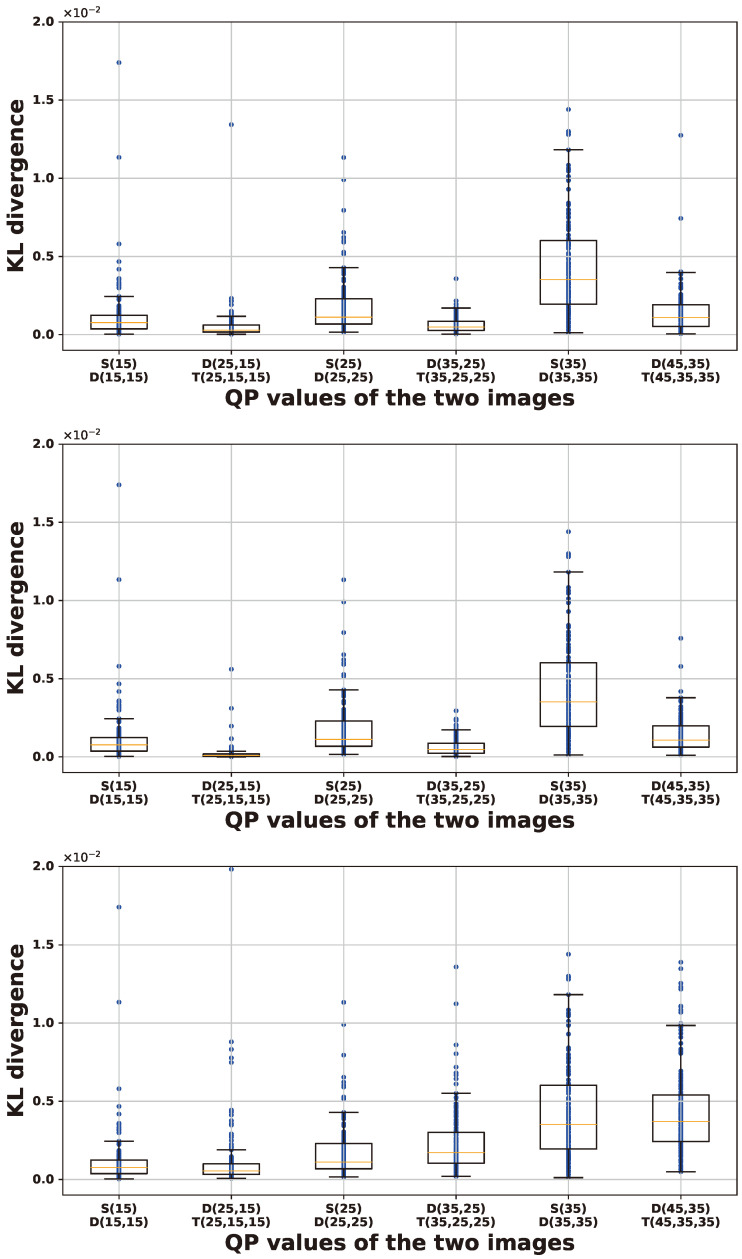
Box-and-whisker diagram of KL divergence for chrominance intra-prediction direction. (**Top**: $QP_{1}>QP_{2}$; **Middle**: $QP_{1}=QP_{2}$; **Bottom**: $QP_{1}<QP_{2}$).

Figure 6, Figure 7 and Figure 8 illustrate box-and-whisker plots showing the distribution of KL divergence between input images and their recompressed counterparts for PU size, luminance intra-prediction direction, and chrominance intra-prediction direction, respectively. The *x*-axis represents the QP values used to generate the input and recompressed images, plotted from left to right in the order of single-compressed image (S) vs. double-compressed image (D) and double-compressed image (D) vs. triple-compressed image (T). The *y*-axis represents the KL divergence, computed by encoding 150 different images. Each plot presents the results for each QP scenario from left to right as follows: (1) $QP_{1}$ is larger than $QP_{2}$, (2) $QP_{1}$ is equal to $QP_{2}$, and (3) $QP_{2}$ is larger than $QP_{1}$. The bottom of the box indicates the first quartile (Q1), and the top indicates the third quartile (Q3). The yellow line inside the box represents the median (Q2) of the data. The whisker extending from the bottom of the box shows the range from Q1 to 1.5 times the interquartile range (IQR), and the whisker extending from the top of the box shows the range from Q3 to 1.5 times the IQR. Data points beyond these ranges are considered outliers. The results in scenarios (1) and (2) show that the variation in KL divergence between double-compressed and triple-compressed images is usually smaller than in KL divergence between single-compressed and double-compressed images. This is evident from the higher Q2 values and wider IQRs of the scenarios in most cases. On the other hand, scenario (3) does not show a clear difference in KL divergence, which makes it difficult for the classifier to identify the double-compressed images.

### 4.3. Combining Feature Vectors

The above analysis compared the energy ratio of the entire MAE difference plot to the energy of the right-hand side of $QP_{2}$. By training the classifier to learn this ratio, we obtained an algorithm equivalent to the model used in [17] without setting a specific threshold. Furthermore, we overcame the model’s limitations in [17] by observing that the more times images are compressed, the smaller the change in coding coefficients between them. The feature vectors reflecting the results of the analysis are as follows:

1. The ratio of the MAE difference energy to the total MAE difference energy on the right side of $QP_{2}$.
2. The histograms of PU size, luminance intra-prediction direction, and chrominance intra-prediction direction.
3. The KL divergence for the variation between images concerning PU size, luminance intra-prediction direction, and chrominance intra-prediction direction.
4. The QP value used for the last encoding.

From the information presented above, a 44-dimensional feature vector per image pair was created for a single-compressed image and its recompressed image or a double-compressed image and its recompressed image pair. This feature vector was input to an SVM classifier with a linear kernel to train and test a model for classifying single- and double-compressed images. We used Scikit-learn’s Support Vector Classifier (SVC) with a linear kernel. Min-Max scaling was applied to the dataset to ensure that all features contributed equally to the SVM classifier. Each feature was scaled to a range between 0 and 1. To determine the optimal value of the regularization parameter *C*, we performed a grid search over the following set of *C* values: {0.01, 0.1, 1, 10, 100, 1000, 2000, 3000, 4000, 5000}. The model’s performance was evaluated using cross-validation on the training set, and the *C* value that yielded the highest cross-validation accuracy was selected as the optimal parameter for our final model.

## 5. Experimental Results

This section describes the experimental validation of the proposed model. We outline the procedure for creating the dataset and compare the accuracy of our double compression detector with conventional approaches in different scenarios. We also investigate the robustness of our method against images generated using different encoding tools.

In the evaluation process, single-compressed images are labeled as negative and double-compressed images as positive. There is no overlap in image content between the training and test data. For clarity, the single-compressed image and its recompressed version are collectively described as a single-compressed image pair, and the double-compressed image and its recompressed version are described as a double-compressed image pair.

In Section 5.2, Section 5.3, Section 5.4 and Section 5.5, we compare our experimental results with those obtained on the same dataset by the method in [17].

### 5.1. Dataset

The dataset for our experiments comprises 300 TIF images featuring various indoor and outdoor scenes, including landscapes, buildings, objects, and nature. These images were captured using three camera models (Nikon D90, Nikon D40, and Nikon D7000) and were selected from the highly cited RAISE forensic dataset [31].

We included all 76 available images from the Nikon D40. The remaining 232 images were chosen from the Nikon D90 (116 images) and Nikon D7000 (116 images), making up a total of 308 images. From these 308 images, we selected 300 for our dataset. The overall breakdown of image content in the dataset is as follows: 70 buildings, 66 indoor scenes, 53 outdoor scenes, 41 objects, 40 nature scenes, and 38 landscapes. Some images contain multiple types of content. The image data can be accessed at [32].

While the primary focus of our experiment is on detecting double compression rather than specific content or camera models, we ensured a diverse selection to cover various scenarios and improve the generalizability of our results.

All images were cropped to a 3:2 aspect ratio and resized to 1200 × 800 pixels using the INTER-AREA interpolation algorithm from the OpenCV library to prevent aliasing. Finally, all images were saved in PNG format.

In this study, we encode and decode images using the open-source HEIF implementation, libheif [33]. Specifically, a PNG image was initially encoded at $QP_{2}$ to generate a single-compressed image. Subsequently, this single-compressed image was recompressed at $QP_{2}$ to produce a recompressed single-compressed image. Similarly, the process of generating double-compressed images involved encoding a PNG image at $QP_{1}$ and recompressing it at $QP_{2}$, resulting in a double-compressed image. This double-compressed image was then recompressed at $QP_{2}$ to create a recompressed double-compressed image. The $QP_{1}$ values were selected from the set {10, 15, 20, 25, 30, 32, 35, 40, 45, 50}, while the $QP_{2}$ values were chosen from {5, 10, 16, 20, 24, 27, 32, 39, 42, 45}.

The maximum CTU size was set to 64. HEIF encoding utilized x265, a popular open-source HEVC encoder offering ten predefined preset options balancing encoding speed and image quality. This study employed the default (0) “medium” preset. It is important to note that x265 typically applies the input QP to the P-slice and adjusts the QP of the I-slice using an offset. To ensure the direct impact of the input QP on our HEIF still images, the offset value was adjusted to zero using the related libheif command (./heif-enc -p x265:ipratio = 1.0).

The above procedure generated 3000 single-compressed image pairs (3000 single-compressed images and 3000 recompressed single-compressed images.) The encoding process for the double-compressed image pairs was performed for each of three scenarios: (a) when $QP_{1}$ exceeds $QP_{2}$, (b) when $QP_{1}$ equals $QP_{2}$, and (c) when $QP_{2}$ exceeds $QP_{1}$. In scenario (a), 17,100 double-compressed and 17,100 recompressed double-compressed images were generated. In scenario (b), 3000 double-compressed and 3000 recompressed double-compressed images were generated. In scenario (c), 11,700 double-compressed and 11,700 recompressed double-compressed images were generated.

### 5.2. Performance Evaluation on Double Compression Detection for Mixed QP Scenario

To assess the performance of the double compression classifier on a test dataset containing images generated in all QP scenarios, we performed a 10-fold cross-validation on a dataset consisting of 3000 single-compressed image pairs and 3000 double-compressed image pairs. The double-compressed image pairs were selected equally from the three QP scenarios (1000 each) and added to the dataset. In every fold of the training process, 600 image pairs (300 single-compressed image pairs and 300 double-compressed image pairs) were selected from the entire dataset as a test dataset. The remaining 5400 image pairs were split into 4800 for training and 600 for validation. The best model was calculated by varying the candidate values of the regularization parameter *C*, and its generalization performance was evaluated using the best model and test data.

**Figure 9:**
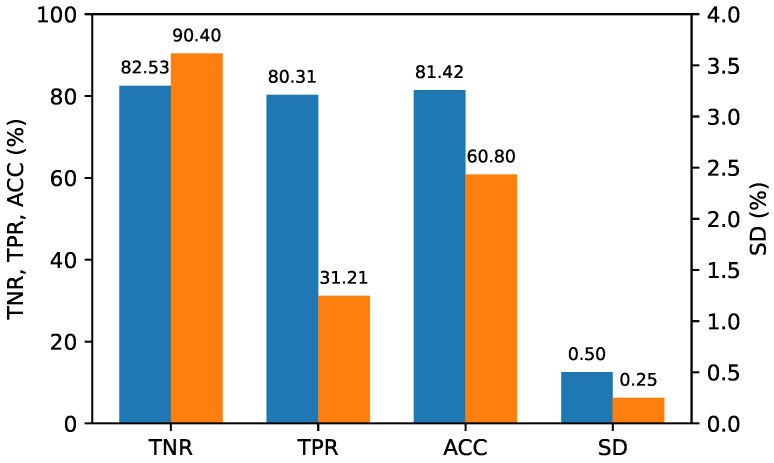
Performance for mixed QP scenarios. (**Blue**: The proposed method; **Orange**: Ref. [17]).

Figure 9 shows the average performance of ten evaluations. For evaluation, we considered the true positive rate (TPR), true negative rate (TNR), and accuracy (ACC). TPR, indicating the proportion of actual positive samples correctly predicted, was calculated as $\mathrm{TPR}=\frac{\mathrm{TP}}{\mathrm{TP}+\mathrm{FN}}$, while TNR, representing the proportion of actual negative samples correctly predicted, was calculated as $\mathrm{TNR}=\frac{\mathrm{TN}}{\mathrm{TN}+\mathrm{FP}}$. We also calculated accuracy as $\mathrm{ACC}=\frac{\mathrm{TPR}+\mathrm{TNR}}{2}$. Standard deviations (SD) were also computed for these metrics. Our model achieved an accuracy of 81% and clearly outperformed the model in [17], which only considered coding ghosts.

### 5.3. Performance Evaluation on Double Compression Detection for Each QP Scenario

**Figure 10:**
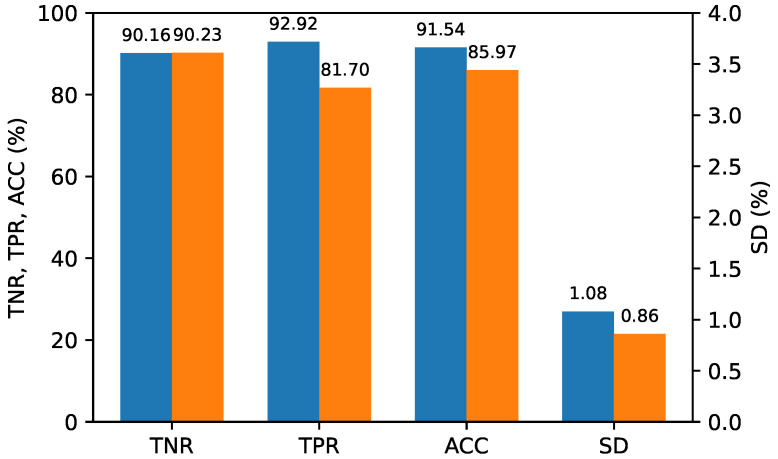
Performance for scenario ($QP_{1}>QP_{2})$. (**Blue**: The proposed method; **Orange**: Ref. [17]).

**Figure 11:**
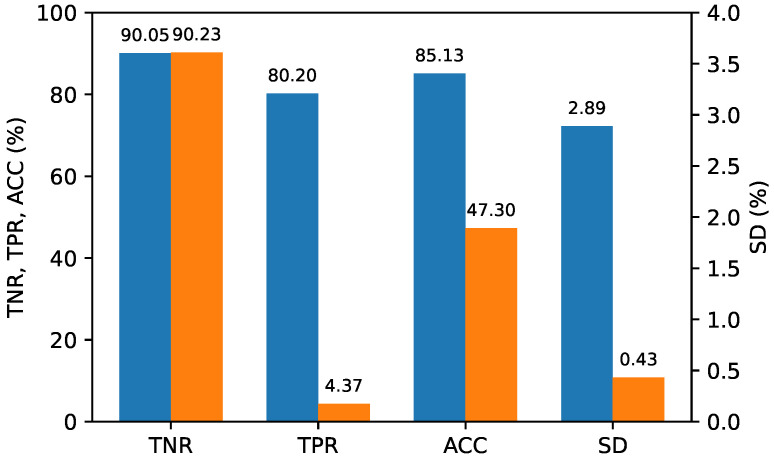
Performance for scenario ($QP_{1}=QP_{2})$. (**Blue**: The proposed method; **Orange**: Ref. [17]).

**Figure 12:**
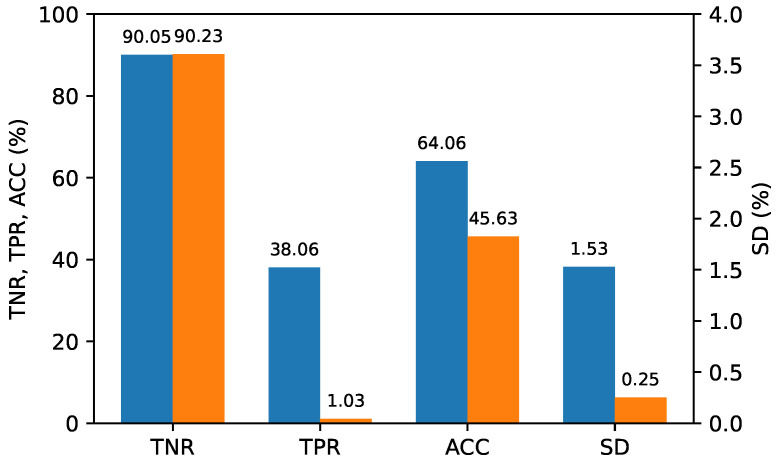
Performance for scenario ($QP_{1}<QP_{2})$. (**Blue**: The proposed method; **Orange**: Ref. [17]).

To assess the performance of the double compression classifier for each QP scenario, we evaluated the test datasets containing the feature vectors of the double-compressed image pairs encoded in three different QP scenarios using a single classifier. Specifically, 300 single-compressed image pairs were randomly extracted from the entire dataset and combined with 300 double-compressed image pairs extracted for each of the three QP (quantization parameter) scenarios, creating test datasets of 600 image pairs for each QP scenario. The entire dataset of 5400 image pairs was divided into 4800 for training and 600 for validation, using 9-fold cross-validation. The best model was saved while varying the C-values, and its generalization performance was evaluated on the three test datasets. To compare the performance of the proposed method and [17] clearly, we fixed the TNR at 90%, or as close to it as possible, and the TPR was calculated accordingly. This evaluation was repeated ten times, and the average performance was calculated. Figure 10, Figure 11 and Figure 12 report the average performance for each QP scenario, respectively, showing that our proposed method outperformed Ref. [17] in all QP scenarios.

### 5.4. Performance Evaluation on Double Compression Detection for Each QP Combination

We evaluated the performance of the double compression classifier for each QP combination of double-compressed images. Specifically, a test dataset was constructed by extracting 30 single-compressed image pairs and 30 double-compressed image pairs for each QP combination from the entire dataset. There were 106 QP combination patterns: 57 for scenario (a) when $QP_{1}$ exceeds $QP_{2}$, 10 for scenario (b) when $QP_{1}$ equals $QP_{2}$, and 39 for scenario (c) when $QP_{2}$ exceeds $QP_{1}$. To ensure fairness, the single-compressed images used for each test dataset were encoded using the final QP values applied in the double-compressed images. For instance, when evaluating double-compressed images with $QP_{1}$ = 30 and $QP_{2}$ = 20, the single-compressed images included in the test data were encoded with QP = 20. This approach ensures that the final QP value is consistent across both single- and double-compressed images, preventing any distinction based on QP values and maintaining fairness in the analysis.

The entire dataset of 5400 image pairs was divided into 4800 for training and 600 for validation, using 9-fold cross-validation. The best model was saved while varying the *C* candidate values during the training phase, and its generalization performance was evaluated on each test dataset. This evaluation procedure was repeated 10 times to ensure robustness, and the average performance over these iterations was calculated.

We calculated the area under the curve (AUC) of the receiver operating characteristic (ROC) curve to compare the methods in [17] and our proposed method for each test dataset. AUC is a threshold-independent standard metric for evaluating a model’s performance. Table 2, Table 3 and Table 4 compare the mean AUC of [17] and our new approach for each QP scenario.

In scenario (a), the weakness in [17] was its inability to correctly identify double-compressed images with a QP difference of 5 or less. However, the new model compensated for this weakness. The model in [17] did not work well in scenarios (b) and (c). Our new approach achieved high performance in scenario (b) and also outperformed [17] in scenario (c).

### 5.5. Performance Evaluation on Double Compression Detection with Different Software

In this subsection, we assess the robustness of our proposed method when creating HEIF images using another image editing software, GIMP. Let S1 be the software used for single compression and S2 be the software used for double compression. We selected 40 TIF images from the entire dataset, converted them to PNG format, and used 30 of them to create 90 single-compressed images and the remaining 10 to create 90 double-compressed images for each of three different software combinations: (A) (S1, S2) = (GIMP, GIMP), (B) (S1, S2) = (libheif, GIMP), and (C) (S1, S2) = (GIMP, libheif). However, as GIMP uses quality factor (QF) instead of QP for encoding, experiments were conducted with new encoding parameters for each of (A), (B), and (C). PNG images were encoded with $QP_{2}$ or $QF_{2}$ to create single-compressed images, and PNG images were encoded with $QP_{1}$ or $QF_{1}$ and then recompressed with $QP_{2}$ or $QF_{2}$ to create double-compressed images. The encoding parameters in (A) were selected from {90, 85, 70} for $QF_{1}$ and {90, 85, 70} for $QF_{2}$. In (B), $QF_{2}$ was chosen from {90, 85, 70} and $QP_{1}$ from {2, 4, 12}. In (C), $QP_{2}$ was selected from {2, 4, 12} and $QF_{1}$ from {90, 85, 70}. Note that QF{90, 85, 70} is equivalent to QP{2, 4, 12}, and QP is used when comparing the encoding parameters in scenarios (B) and (C).

To evaluate the performance of the double compression classifier in different software combinations, we calculated AUC for each QP combination for each datum. A test dataset was constructed by extracting 10 single-compressed image pairs and 10 double-compressed image pairs for each software combination from the entire dataset. To ensure fairness, the single-compressed images used for each test dataset were only encoded with the final QP values used for the double-compressed images. We created a dataset consisting of 2400 single-compressed and 2400 double-compressed image pairs, excluding the images used to generate the test data, for 4800 image pairs. The dataset was split into 4320 for training and 480 for validation using a 10-fold cross-validation. The model with the best performance was saved while varying the C candidate values. Table 5, Table 6 and Table 7 display the average performance of 10 evaluations for software combinations (A), (B), and (C).

When the HEIF image was generated by (A), the double-compressed image was successfully detected if $QF_{1}$ was smaller or the same as $QF_{2}$. Using the same software ensures high consistency in the compression algorithm, making the characteristics of double compression more distinct and, thus, easier to detect. On the other hand, the detection accuracy in (B) and (C) was better when $QP_{1}$ was larger than $QP_{2}$. Due to differences in compression algorithms between different software, the compression characteristics vary, making it more difficult to detect double compression, resulting in lower detection accuracy than (A). As a result of the observations, images encoded with libheif are more easily distinguishable in terms of MAE difference than those encoded with GIMP. Therefore, the accuracy of (C) is slightly higher than that of (B). Due to the different encoding algorithms of libheif and GIMP, the detection accuracy in (B) and (C) was better when $QP_{1}$ was larger than $QP_{2}$, while slightly lower than in (A) when they were equal.

## 6. Conclusions

In this work, we extended our previous research and proposed a lightweight image classifier that detects double compression in HEIF images by extracting and learning encoding information from the input image and its recompressed image. The proposed method achieved an accuracy of 81% by clearly outperforming the model in [17], which just reaches 60% of accuracy. To the best of our knowledge, our work is the first to address double compression detection in HEIF images. Our new approach addresses the limitations imposed by quantization history in double-compressed images. In future work, we will take into account the local image content to improve the performance of the detector. Indeed, our proposed method extracts encoding information from all regions in the image, including areas that do not contribute to double compression detection. Moreover, we will consider extending the analysis to identify forged areas in the image, for example, by running the double detection method in a block-wise fashion and detecting the presence of both double-compressed and single-compressed blocks within the same image.

## Figures and Tables

**Table 1:** The maximum and minimum sizes of the four processing units.

Unit	Max	Min
CTU	64 × 64	16 × 16
CU	64 × 64	8 × 8
PU	64 × 64	4 × 4
TU	32 × 32	4 × 4

**Table 2:** Average AUC for scenario ($QP_{1}>QP_{2}$). (**Top**: Ref. [17]; **Bottom**: The proposed method).

		$QP_{2}$									
$QP_{1}$		5	10	16	20	24	27	32	39	42	45
	10	0.53	-	-	-	-	-	-	-		
	15	0.91	0.43	-	-	-	-	-	-	-	-
	20	0.94	0.84	0.41	-	-	-	-	-	-	-
	25	0.98	0.96	0.82	0.73	0.55	-	-	-	-	-
	30	1.0	1.0	0.99	0.99	1.0	0.91	-	-	-	-
	32	1.0	1.0	0.99	1.0	1.0	0.99	-	-	-	-
	35	1.0	1.0	1.0	1.0	1.0	1.0	0.95	-	-	-
	40	1.0	1.0	1.0	1.0	1.0	1.0	1.0	0.82	-	-
	45	1.0	1.0	1.0	1.0	1.0	1.0	1.0	1.0	0.93	-
	50	1.0	1.0	1.0	1.0	1.0	1.0	1.0	1.0	1.0	0.99
		$QP_{2}$									
$QP_{1}$		5	10	16	20	24	27	32	39	42	45
	10	0.98	-	-	-	-	-	-	-		
	15	1.0	0.9	-	-	-	-	-	-	-	-
	20	1.0	0.93	0.94	-	-	-	-	-	-	-
	25	1.0	0.96	0.98	0.98	0.95	-	-	-	-	-
	30	0.99	0.99	1.0	1.0	0.99	0.96	-	-	-	-
	32	0.99	0.98	1.0	1.0	1.0	0.99	-	-	-	-
	35	1.0	0.99	1.0	1.0	1.0	1.0	0.94	-	-	-
	40	1.0	0.99	1.0	1.0	1.0	1.0	1.0	0.93	-	-
	45	0.99	0.98	1.0	1.0	1.0	1.0	1.0	0.99	0.95	-
	50	0.99	0.98	0.99	1.0	1.0	1.0	1.0	1.0	0.99	0.96

**Table 3:** Average AUC for scenario ($QP_{1}=QP_{2}$). (**Top**: Ref. [17]; **Bottom**: The proposed method).

		$QP_{2}$									
$QP_{1}$		5	10	16	20	24	27	32	39	42	45
	5	0.2	-	-	-	-	-	-	-		
	10	-	0.3	-	-	-	-	-	-	-	-
	16	-	-	0.29	-	-	-	-	-	-	-
	20	-	-	-	0.36	-	-	-	-	-	-
	24	-	-	-	-	0.43	-	-	-	-	-
	27	-	-	-	-	-	0.34	-	-	-	-
	32	-	-	-	-	-	-	0.42	-	-	-
	39	-	-	-	-	-	-	-	0.57	-	-
	42	-	-	-	-	-	-	-	-	0.59	-
	45	-	-	-	-	-	-	-	-	-	0.62
		$QP_{2}$									
$QP_{1}$		5	10	16	20	24	27	32	39	42	45
	5	0.99	-	-	-	-	-	-	-		
	10	-	0.93	-	-	-	-	-	-	-	-
	16	-	-	0.98	-	-	-	-	-	-	-
	20	-	-	-	0.98	-	-	-	-	-	-
	24	-	-	-	-	0.95	-	-	-	-	-
	27	-	-	-	-	-	0.92	-	-	-	-
	32	-	-	-	-	-	-	0.9	-	-	-
	39	-	-	-	-	-	-	-	0.93	-	-
	42	-	-	-	-	-	-	-	-	0.91	-
	45	-	-	-	-	-	-	-	-	-	0.89

**Table 4:** Average AUC for scenario ($QP_{1}<QP_{2}$). (**Top**: Ref. [17]; **Bottom**: The proposed method).

		$QP_{2}$							
$QP_{1}$		16	20	24	27	32	39	42	45
	10	0.48	0.49	0.51	0.51	0.52	0.51	0.5	0.52
	15	0.36	0.49	0.49	0.51	0.53	0.52	0.5	0.53
	20	-	-	0.48	0.54	0.54	0.51	0.52	0.53
	25	-	-	-	0.54	0.59	0.55	0.53	0.53
	30	-	-	-	-	0.58	0.57	0.57	0.55
	32	-	-	-	-	-	0.62	0.58	0.56
	35	-	-	-	-	-	0.59	0.58	0.59
	40	-	-	-	-	-	-	0.69	0.62
		$QP_{2}$							
$QP_{1}$		16	20	24	27	32	39	42	45
	10	0.74	0.71	0.66	0.63	0.6	0.55	0.55	0.53
	15	0.98	0.84	0.81	0.76	0.64	0.62	0.56	0.54
	20	-	-	0.78	0.83	0.76	0.66	0.6	0.59
	25	-	-	-	0.88	0.8	0.74	0.65	0.61
	30	-	-	-	-	0.85	0.76	0.74	0.63
	32	-	-	-	-	-	0.82	0.73	0.65
	35	-	-	-	-	-	0.78	0.74	0.66
	40	-	-	-	-	-	-	0.83	0.67

**Table 5:** Average AUC for software combination (GIMP, GIMP). (**Top**: Ref. [17]; **Bottom**: The proposed method).

		$QF_{2}$ ($QP_{2}$)		
$QF_{1}$ ($QP_{1}$)		90 (2)	85 (4)	70 (12)
	90 (2)	0.26	0.31	0.4
	85 (4)	0.29	0.3	0.39
	70 (12)	0.52	0.45	0.38
		$QF_{2}$ ($QP_{2}$)		
$QF_{1}$ ($QP_{1}$)		90 (2)	85 (4)	70 (12)
	90 (2)	0.91	0.59	0.6
	85 (4)	0.76	0.91	0.53
	70 (12)	0.96	0.97	0.96

**Table 6:** Average AUC for software combination (libheif, GIMP). (**Top**: Ref. [17]; **Bottom**: The proposed method).

		$QF_{2}$ ($QP_{2}$)		
$QP_{1}$ ($QF_{1}$)		90 (2)	85 (4)	70 (12)
	2 (90)	0.24	0.33	0.42
	4 (85)	0.29	0.24	0.42
	12 (70)	0.75	0.67	0.35
		$QF_{2}$ ($QP_{2}$)		
$QP_{1}$ ($QF_{1}$)		90 (2)	85 (4)	70 (12)
	2 (90)	0.6	0.53	0.63
	4 (85)	0.81	0.54	0.67
	12 (70)	0.97	0.92	0.88

**Table 7:** Average AUC for software combination (GIMP, libheif). (**Top**: Ref. [17]; **Bottom**: The proposed method).

		$QP_{2}$ ($QF_{2}$)		
$QF_{1}$ ($QP_{1}$)		2 (90)	4 (85)	12 (70)
	90 (2)	0.18	0.22	0.36
	85 (4)	0.16	0.27	0.36
	70 (12)	0.33	0.42	0.33
		$QP_{2}$ ($QF_{2}$)		
$QF_{1}$ ($QP_{1}$)		2 (90)	4 (85)	12 (70)
	90 (2)	0.62	0.74	0.47
	85 (4)	0.68	0.87	0.56
	70 (12)	0.98	1.0	0.89

## Data Availability

The dataset presented in the study is available on GitHub at https://git.lesc.dinfo.unifi.it/yoshihisa/TIFF_image. It was originally extracted from the RAISE dataset (http://loki.disi.unitn.it/RAISE/index.php) created by the Multimedia Signal Processing and Understanding Lab at the University of Trento, Italy, and has been processed as described in the study.

## Abbreviations

The following abbreviations are used in this manuscript:

QP	Quantization Parameter
QF	Quality Factor
DCT	Discrete Cosine Transform
GOP	Group of Pictures
CTU	Coding Tree Unit
CU	Coding Unit
PU	Prediction Unit
TU	Transform Unit
MAE	Mean Absolute Error
KLD	Kullback–Leibler Divergence
TPR	True Negative Rate
TPR	True Positive Rate
ACC	Accuracy
SD	Standard Deviation
AUC	Area Under the Curve

## References

[1] Verdoliva L. (2020). Media forensics and deepfakes: An overview. IEEE J. Sel. Top. Signal Process..

[2] Wallace G.K. (1992). The JPEG still picture compression standard. IEEE Trans. Consum. Electron..

[3] Lin Z., He J., Tang X., Tang C.K. (2009). Fast, automatic and fine-grained tampered JPEG image detection via DCT coefficient analysis. Pattern Recognit..

[4] Barni M., Bondi L., Bonettini N., Bestagini P., Costanzo A., Maggini M., Tondi B., Tubaro S. (2017). Aligned and non-aligned double JPEG detection using convolutional neural networks. J. Vis. Commun. Image Represent..

[5] Fu D., Shi Y.Q., Su W. A generalized Benford’s law for JPEG coefficients and its applications in image forensics. Proceedings of the Security, Steganography, and Watermarking of Multimedia Contents IX, SPIE.

[6] Li B., Shi Y.Q., Huang J. Detecting doubly compressed JPEG images by using mode based first digit features. Proceedings of the 2008 IEEE 10th Workshop on Multimedia Signal Processing.

[7] Amerini I., Becarelli R., Caldelli R., Del Mastio A. Splicing forgeries localization through the use of first digit features. Proceedings of the 2014 IEEE International Workshop on Information Forensics and Security (WIFS).

[8] Farid H. (2009). Exposing digital forgeries from JPEG ghosts. IEEE Trans. Inf. Forensics Secur..

[9] Luo W., Qu Z., Huang J., Qiu G. A novel method for detecting cropped and recompressed image block. Proceedings of the 2007 IEEE International Conference on Acoustics, Speech and Signal Processing-ICASSP’07.

[10] Ye S., Sun Q., Chang E.C. Detecting digital image forgeries by measuring inconsistencies of blocking artifact. Proceedings of the 2007 IEEE International Conference on Multimedia and Expo.

[11] Li W., Yuan Y., Yu N. (2009). Passive detection of doctored JPEG image via block artifact grid extraction. Signal Process..

[12] Qu Z., Luo W., Huang J. A convolutive mixing model for shifted double JPEG compression with application to passive image authentication. Proceedings of the 2008 IEEE International Conference on Acoustics, Speech and Signal Processing.

[13] Bianchi T., Piva A. (2011). Detection of nonaligned double JPEG compression based on integer periodicity maps. IEEE Trans. Inf. Forensics Secur..

[14] High Efficiency Image File Format. https://developer.apple.com/videos/play/wwdc2017/513/.

[15] McKeown S., Russell G. Forensic considerations for the high efficiency image file format (heif). Proceedings of the 2020 International Conference on Cyber Security and Protection of Digital Services (Cyber Security).

[16] Baracchi D., Iuliani M., Nencini A.G., Piva A. (2021). Facing image source attribution on iPhone X. Proceedings of the Digital Forensics and Watermarking: 19th International Workshop, IWDW 2020.

[17] Furushita Y., Fontani M., Bressan M., Bianchi S., Piva A., Ramponi G. (2024). Double Compression Detection of HEIF Images Using Coding Ghosts. Proceedings of the Ninth International Congress on Information and Communication Technology: ICICT 2024.

[18] Xu Q., Sun T., Jiang X., Dong Y. (2017). HEVC double compression detection based on SN-PUPM feature. Proceedings of the Digital Forensics and Watermarking: 16th International Workshop, IWDW 2017.

[19] Jiang X., He P., Sun T., Wang R. (2019). Detection of double compressed HEVC videos using GOP-based PU type statistics. IEEE Access.

[20] He P., Li H., Wang H., Wang S., Jiang X., Zhang R. (2020). Frame-wise detection of double HEVC compression by learning deep spatio-temporal representations in compression domain. IEEE Trans. Multimed..

[21] Huang M., Wang R., Xu J., Xu D., Li Q. (2015). Detection of double compression for HEVC videos based on the co-occurrence matrix of DCT coefficients. Proceedings of the International Workshop on Digital Watermarking.

[22] Liang X., Li Z., Yang Y., Zhang Z., Zhang Y. (2018). Detection of double compression for HEVC videos with fake bitrate. IEEE Access.

[23] Jiang X., Xu Q., Sun T., Li B., He P. (2019). Detection of HEVC double compression with the same coding parameters based on analysis of intra coding quality degradation process. IEEE Trans. Inf. Forensics Secur..

[24] Sze V., Budagavi M., Sullivan G.J. (2014). High efficiency video coding (HEVC). Integrated Circuit and Systems, Algorithms and Architectures.

[25] Sullivan G.J., Ohm J.R., Han W.J., Wiegand T. (2012). Overview of the high efficiency video coding (HEVC) standard. IEEE Trans. Circuits Syst. Video Technol..

[26] Bestagini P., Milani S., Tagliasacchi M., Tubaro S. Video codec identification extending the idempotency property. Proceedings of the European Workshop on Visual Information Processing (EUVIP).

[27] Lukáš J., Fridrich J. Estimation of primary quantization matrix in double compressed JPEG images. Proceedings of the Digital Forensic Research Workshop.

[28] Bianchi T., De Rosa A., Fontani M., Rocciolo G., Piva A. Detection and classification of double compressed MP3 audio tracks. Proceedings of the First ACM Workshop on INFORMATION Hiding and Multimedia Security.

[29] heic2hevc. https://github.com/yohhoy/heic2hevc.

[30] HM Software. https://hevc.hhi.fraunhofer.de.

[31] Dang-Nguyen D.T., Pasquini C., Conotter V., Boato G. Raise: A raw images dataset for digital image forensics. Proceedings of the 6th ACM Multimedia Systems Conference.

[32] TIFF Images Used for Experiment. https://git.lesc.dinfo.unifi.it/yoshihisa/TIFF_image.

[33] Libheif. https://github.com/strukturag/libheif.

